# Phenotypic variability of *CCDC103 *mutation in British Pakistani children with Primary Ciliary Dyskinesia (PCD)

**DOI:** 10.1186/2046-2530-4-S1-P61

**Published:** 2015-07-13

**Authors:** E Robson, E Moya, T Burgoyne, P Chetcuti, M Dixon, R Hirst, C Hogg, H Mitchison, C O'Callaghan, A Onoufriadis, M Patel, A Rutman, E Sheridan, A Shoemark

**Affiliations:** 1 Department of Respiratory Paediatrics, Leeds/ Bradford Primary Ciliary Dyskinesia Regional Unit, Leeds, UK; 2 Leeds Institute for Molecular Medicine, Wellcome Trust Brenner Building, St James's Hospital, Leeds, UK; 3 Centre for PCD Diagnosis and Research, Department of Infection, Immunity and Inflammation, University of Leicester, Leicester Royal Infirmary, Leicester, UK; 4 Genetics and Genomics Medicine, University College London Institute of Child Health, London, UK; 5 Department of Respiratory Paediatrics, Primary Ciliary Dyskinesia Centre, Royal Brompton and Harefield Foundation Trust, London, UK

## Objectives

PCD is an autosomal recessive condition that affects the structure and function of motile cilia in the respiratory tract, middle ear and reproductive organs. The estimated prevalence is 1:15,000, but as high as 1:2265 in the British Asian population.

Mutations in the *CCDC103 *gene have recently been identified as PCD disease-causing in Pakistani individuals. It is found to be an essential gene for dynein arm assembly and ciliary motility.

## Methods

We present eleven British Pakistani children found to be homozygous for a missense mutation in *CCDC103*, resulting in the amino acid substitution His154Pro.

## Results

Nasal Nitric Oxide screening test results were normalised, mean 290ppb (range 22-857), compared to usual values in PCD (<100ppb). Ciliary beat frequency was also often in the normal range 10.6Hz (range 0- 16.3).

Seven had a defect of the ciliary inner and outer dynein arms demonstrated in ciliated nasal cells by electron microscopy. This defect was partial and distinct from the near complete absence of dynein arms seen in children with mutations in *LRRC6 *and *ZMYND10*.

A further four children (3 siblings) presented with a phenotype suggestive of PCD but electron microscopy studies were inconclusive on repeat testing. Genetic testing revealed the same *CCDC103 *homozygous mutations, making the diagnosis of PCD possible based on genetic analysis.

## Conclusion

We report a high prevalence of the *CCDC103 *His154Pro mutation in the British Asian PCD community and the phenotypic variability of *CCDC103 *in order to raise awareness of the potential benefit of genetic testing as a diagnostic aid in non-typical PCD cases.

